# *COMT* genotype and non-recovery after a whiplash injury in a Northern European population

**DOI:** 10.1186/s12891-017-1810-z

**Published:** 2017-12-01

**Authors:** Eric Rydman, Erika Comasco, H. Pettersson, L. Oreland, S. Ponzer, C. Ottosson

**Affiliations:** 1 Department of Clinical Science and Education, Södersjukhuset, Karolinska Institutet, -118 83 Stockholm, SE Sweden; 2 Department of Neuroscience, Uppsala University, Uppsala, Sweden

**Keywords:** Whiplash, Wad, COMT, Catechol O-methyltransferase, Pain, Genetics, Recovery

## Abstract

**Background:**

The *COMT* (Catechol-O-Methyl Transferase) gene may influence a person’s vulnerability to develop long-term pain and some *COMT* single nucleotide polymorphisms (SNPs) may associate with patterns of acute or chronic pain. Many patients with whiplash-associated disorders (WADs) suffer from long-term pain and other related symptoms, but it is less known if genetic factors play a role in the recovery process. The primary aim of this study was to evaluate whether self-reported non-recovery, including pain, was related to *COMT* genotype in patients with WAD. The secondary aim was to investigate whether or not background factors, including mental health, were related to genotype and non-recovery.

**Methods:**

A total of 133 patients with neck pain after a whiplash trauma were included. Background factors were collected and blood samples were taken during the acute phase after the accident. DNA was isolated from blood and used to genotype the SNPs rs6269, rs4633, rs4818 and rs4680 in the *COMT* gene; additionally haplotypes were estimated and haplogenotypes inferred. The patients were followed up after 12 months and asked to rate their recovery including pain, mental health and quality of life.

**Results:**

The overall reported non-recovery rate at 12 months was 44% with no significant differences in distribution of the *COMT* haplotypes. High levels of self-reported pain (OR 7.2) and anxiety (OR 4.4) after the accident were associated with non-recovery, but not related to the haplotypes. None of the other background factors were related to the haplotypes or non-recovery.

**Conclusion:**

No association between self-reported non-recovery or pain levels and *COMT* haplotypes in patients with acute whiplash injuries could be detected. Independent replications are necessary to discard the hypothesis that *COMT* haplotypes do not influence non-recovery or pain levels in patients with acute whiplash injuries. High levels of initial pain and anxiety were associated with non-recovery, thereby confirming previously published reports.

## Background

Despite several attempts to understand the differences in the prognosis of recovery after a whiplash trauma, the biological mechanisms remain unexplained. The main symptom in patients with a whiplash-associated disorder (WAD) is long-lasting pain [1]. Initial pain sensations after the trauma seem to be the best prognostic factors for the outcome in patients suffering from WAD [2–5].

It has been suggested that genetic predisposition might play a role in the pathogenesis of WAD. In the field of pain genetics, the catechol-*O*-methyltransferase (*COMT*) gene is one of the most well-studied ones [6]. It has been suggested that polymorphisms within the gene regulate pain perception in healthy subjects [7], as well as in patients with fibromyalgia [8, 9], lower back pain [9, 10] and also acute pain after motor vehicle accidents [11]. Tammimäki and Männistö’s review of the literature indicated that diverging results from published studies call for further investigations, especially in the field of musculoskeletal pain [12].

The *COMT* gene regulates formation of both soluble and membrane-bound protein isoforms. It contains many single nucleotide polymorphisms (SNPs), some of which have been investigated concerning their role in pain regulation. The *COMT* Val158Met SNP, rs4680, is the most frequently investigated variant [9]. The Met/Met genotype of the SNP rs4680 has been associated with several mental diseases [13], as well as fibromyalgia and widespread pain [14]. In addition, the importance of haplotypes has been demonstrated, especially regarding the haplotype that includes rs6269, coding for the soluble form, and rs4633, rs4680 and rs4818, coding for both the soluble and membrane-bound forms. Three common haplotypes based on linkage disequilibrium and pain-related data have been identified [15]. The low pain sensitivity (LPS) haplotype codes for the highest levels of the COMT enzyme activity and has been suggested to mediate for high tolerance of musculoskeletal pain. The average pain sensitivity (APS) haplotype codes for lower enzyme activity and average pain tolerance. The high pain sensitivity (HPS) haplotype is known to code for the lowest levels of the enzyme and the lowest tolerance of pain [15, 16].

While the *COMT* gene contains functional polymorphisms that have been found to influence human pain [17–19], its role in WAD is not known. Because of the relationship between initial pain sensation and recovery in WAD patients [20], it is plausible to hypothesize that *COMT* variations could influence pain regulation and clinical outcome in this patient population.

The primary aim of this study was to evaluate whether or not self-reported non-recovery, including pain, was related to *COMT* genotype in patients with WAD. The secondary aim was to investigate whether or not background factors, including mental health, were related to haplotypes and to non-recovery.

## Methods

### Participants

Patients were enrolled between September 2002 and January 2004. Potentially eligible patients had sustained a whiplash injury less than 24 h before arrival at the emergency department (ED). Exclusion criteria were WAD IV (fractures), age < 15 years, inability to read and understand the Swedish language and impaired cognitive function making the self-report impossible. The medical treatment was administered according to the routine protocols at the hospital. The patients were informed about the study and those who agreed to participate were invited to an out-patient visit for inclusion. The patients were originally part of a randomized controlled trial in which standard treatment was compared with an intervention [21].

A blood sample for the genetic analysis was taken and the participants were asked to fill in questionnaires regarding socio-demographic information, including age (<24, 25–65, >66), gender, educational level (high school or less or university), working status, marital status and ethnic background (Caucasian, Caucasian-Hispanic, Asian, Middle Eastern or African). They were asked to rate their health-related quality of life (HRQoL) the week before the accident (i.e., a retrospective rating) according to the Short Form-36 (SF-36) Health Survey [22]. Visual analogue scales (VAS) were used for ratings of pain and mental status (anxiety and depression) the week before the whiplash injury occurred (i.e., a retrospective rating) and at the time of inclusion, with 0 indicating minimum and 100 maximum symptoms. The patients also filled in the Hospital Anxiety and Depression (HAD) [23] and the Posttraumatic Stress Disorder (PTSD-10) questionnaires [24]. A score of >10 indicated a state of anxiety or depression on the HAD score [25] and a score of >5 indicated PTSD according to PTSD-10 [26]. These self- reported data did not, however, fulfil the criteria for the medical conditions per se.

### Genetic analysis

Blood was used to isolate DNA using the QIAamp DNA MiniKit, according to the manufacturer’s protocol (http://www.qiagen.com/). The selected polymorphisms in the *COMT* gene were: rs6269, rs4633, rs4818 and rs4680. The *COMT* rs4680 (Val^158^Met) polymorphism was genotyped using the TaqMan® Drug Metabolism Genotyping Assay, according to the manufacturer’s protocol [27]. This assay consists of sequence-specific forward and reverse primers and two TaqMan® MGB probes, each labelled with a FAM™ or VIC® reporter dye at the 5’end and a non-fluorescent quencher at the 3’end of each one. Polymerase chain reaction (PCR) was performed in a 5-μl reaction mixture containing 1 × TaqMan®Universal PCR Master Mix 2.5 μl; 40× TaqMan®Drug Metabolism Genotyping Assay Mix 0.25 μl and 3–20 ng Genomic DNA diluted in H_2_O (Applied Biosystem®). An ABI PRISM®7900HT Sequence Detection System was used to perform allele discrimination PCR reactions according to the following thermal cycler conditions: initial step of 10 min at 95 °C, followed by 50 cycles of denaturation at 15 s, 92 °C and annealing at 90 s, 60 °C. The PCR products were analysed and allele sizes were determined using SDS 2.2 (Applied Biosystem®). On the other hand, a fluorescence-based competitive allele-specific PCR (KASPar) assay (KBioscience®), based on a public genome sequence (www.ensembl.org/), was used to analyse the SNPs rs6269, rs4633 and rs4818. Alleles were determined using SNPviewer2®. χ^2^ test or Fisher’s test was used to investigate deviations from Hardy-Weinberg Equilibrium (HWE). The EM algorithm was computed using SNP & Variation Suite 7 (Golden Helix) to estimate linkage disequilibrium (r^2^) and haplotype blocks. Three common haplotypes were found, as in Diatchenko et al. [15]. LPS was defined as homozygous for the G_C_G_G haplotype (GCGG/GCGG). APS was defined as homozygous for the A_T_C_A haplotype (ATCA/ATCA) and heterozygous for ACCG/GCGG or GCCG/ATCA. HPS was defined as heterozygous for ATCA or ACCG.

An additional categorization of genotypes was determined according to the presence of at least one LPS haplotype [11].

### Outcome measures

The primary outcome measure was self-reported recovery at 12 months, assessed by means of the single question: ‘Do you feel recovered after the injury?’ (Yes/No). Secondary outcome measures were the SF-36 responses (physical and mental score) and pain and mental status measured by visual analogue scales (VAS pain and VAS mental).

### Statistical methods

Nominal variables were tested with chi-square test. The Kruskal-Wallis and Anova were used for comparisons measured on interval and ratio scale variables. The results were regarded as significant if *p* was less than 0.05, two-tailed. The *p*-values were presented without adjustment for multiple comparisons. Logistic regression was used to identify variables associated with being risk factors for non-recovery at 12 months. First, crude associations for each selected variable were studied in univariable models. Secondly, a multivariable model was used to adjust the associations. The results were regarded as significant at *p* < 0.05. The associations are presented as odds ratios (ORs) with 95% confidence intervals [28].

The statistical analysis was performed using SPSS version 20.0 (SPSS, Inc., Chicago, Il. USA).

### Ethics

The study was approved by the Regional Research Ethics Committee Stockholm, Sweden and all patients gave their written informed consent before inclusion (Dnr-240/01).

## Results

A total of 133 patients were enrolled in our study, but the outcome analysis was based on 128 patients because five lacked 12-month follow-up data. The distribution of genotypes and haplotypes are presented in Table 1. Most of the patients had the APS haplotype (102 out of 133).

**Table 1:** Distribution of genotypes and haplotypes for the study population (*n* = 133)

	Genotype	n = 133	%	HWE test *p*-value
SNP				
rs6269	A/A	56	42	χ^2^ = 0.75; *p* = 0.39
	A/G	57	43	
	G/G	20	15	
rs4633	C/C	29	22	χ^2^ = 2.14; *p* = 0.14
	C/T	57	43	
	T/T	47	35	
rs4818	C/C	59	44	χ^2^ = 0.64; *p* = 0.42
	C/G	56	42	
	G/G	18	14	
rs4680	A/A	47	35	χ^2^ = 2.75; *p* = 0.09
	A/G	56	42	
	G/G	30	23	
Haplotype				
LPS	GCGG/GCGG	20	15	
APS	ATCA/ATCA ACCG/GCGG or GCCG/ATCA	102	77	
HPS	ATCA or ACCG	11	8	

As shown in Tables 2, 56% of the patients were females; the mean age was 40 years and most of the patients were of Caucasian origin, had a secondary school education, were working and were non-smokers. There were no differences regarding these background factors and the distribution of LPS, APS and HPS haplotypes (Table 2). Mental health data at inclusion showed that 20.2% of the patients had self-reported symptoms of anxiety and 8.7% had signs of PTSD before the whiplash injury, but there were no significant differences between the different haplotypes and these factors as also shown in Table 2.

**Table 2:** Background data at baseline (n = 133). There were no significant differences in any of the comparisons between the variables

	Total n = 133 (%)	LPS *n* = 20 (%)	APS *n* = 102 (%)	HPS *n* = 11 (%)
Female sex	75 (56)	11 (55)	57 (56)	7 (64)
*Ethnicity*				
Caucasian	114 (86)	17 (15)	88 (77)	9(8)
Caucasian-Hispanic	7 (5)	1 (14)	6 (85)	0 (0)
Asian	2 (1)	1 (50)	0 (0)	1 (50)
Middle Eastern	9 (7)	1 (11)	8 (89)	0 (0)
African	1(1)	0 (0)	0 (0)	1 (100)
*Educational level*				
High school	92 (69)	15 (75)	71 (70)	6 (55)
University	41 (31)	5 (25)	31 (30)	5 (45)
Employed (yes)	109 (82)	15 (75)	85 (83)	9 (82)
Smoker (yes)	34 (26)	5 (26)	27 (28)	2 /18)
*Self-ratings of mental health*	n (%)	n	n	n
HAD depression (yes)	12 (9.8)	2	8	2
HAD anxiety (yes)	24 (20.2)	3	20	1
PTSD (yes)	11 (8.7)	3	7	1
Age (years)	mean (SD)	mean (SD)	mean (SD)	mean (SD)
	40 (13)	43 (18)	40 (13)	41 (15)

**Fig. 1:**
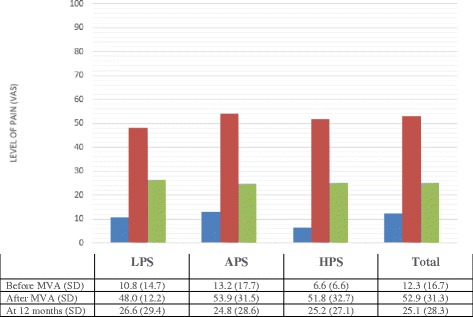
Association between haplotypes reported before the accident, after the accident and at 12- month follow-up and mean levels of pain

No significant differences could be detected either between the distribution of haplotypes and the VAS and SF-36 results before (retrospective rating) or after the accident or at the 12-month follow-up (Fig. 1), (Table 3).

**Table 3:** Pain and mental status ratings on Visual Analogue Scale (VAS) and HRQoL according to SF-36 in relation to haplotype. No significant differences between the haplotype groups

	LPS		APS		HPS		Total	
	Mean	SD	Mean	SD	Mean	SD	Mean	SD
*VAS pain*								
Before	10.85	14,67	13.16	17.73	6.55	6.55	12.26	16.68
After	48.00	31.21	53.94	31.46	51.82	32.66	52.87	31.35
At 12 months	26.59	29.36	24.83	28.57	25.20	27.09	25.14	28.32
*VAS mental health*								
Before	20.40	30.39	11.40	17.11	15.82	19.82	13.13	19.96
After	41.35	33.47	38.19	32.96	32.18	31.47	38.17	32.74
At 12 months	17.12	22.63	16.10	27.73	16.40	21.99	16.28	26.34
*SF-36 physical score*								
Before	89.74	12.34	87.59	15.74	88.18	13.41	87.97	14.98
At 12 months	71.13	27.20	72.54	28.86	73.53	25.84	72.40	28.01
*SF-36 mental score*								
Before	88.12	12.68	85.47	14.46	90.69	3.22	86.32	13.64
At 12 months	73.75	29.58	72.92	26.43	69.83	19.08	72.70	26.00

Fifty-six patients (43.8%) reported non-recovery at 12 months, with no difference in the recovery rate between the different *COMT* haplotypes (LPS 42.1%, APS 43.9% and HPS 45.5%, respectively).

Regression analyses with adjustments for confounding factors (Table 4) showed no significant differences regarding the *COMT* haplotypes in relation to recovery, but high levels of initial pain according to VAS (adjusted OR, 10.4 (3.0–36.6) and anxiety according to HAD ratings (adjusted OR, 3.5 (1.1–11.6) were associated with non-recovery at 12 months.

**Table 4:** Association between the predictor variables and self-reported non-recovery at 12 months after the injury. All predictor variables were measured at baseline. Odds ratios and 95% confidence intervals (CIs)

Predictor variable	Level	Non-recovered/total (n/n) %		Odds ratio (CI) Crude	Odds ratio (CI) Adjusted^a^
Gender	Male	23/54	42.6	1.0 (Reference)	
	Female	33/74	44.6	1.1 (0.5–2.2)	1.2 (0.5–3.0)
Age	<24	6/13	46.2	1.0 (Reference)	
	25–65	49/110	44.5	0.9 (0.3–3.0)	0.7 (0.2–2.7)
	>66	1/5	20.0	0.3 (0.02–3.4)	0.1 (0.0–1.4)
VAS pain after accident	<24	7/35	20.0	1.0 (Reference)	
	25–65	13/37	35.1	2.2 (0.7–6.3)	2.8 (0.9–8.8)
	>65	36/56	64.3	7.2 (2.7–19.4)	10.4 (3.0–36.6)
Anxiety (HAD)	No	35/93	37.6	1.0 (Reference)	
	Yes	16/22	72.7	4.4 (1.6–12.4)	3.5 (1.1–11.6)
Haplotype	LPS	8/19	42.1	1.0 (Reference)	
	APS	43/98	43.9	1.1(0.4–2.9)	0.8 (0.2–2.5)
	HPS	5/11	45.5	1.2 (2.3–5.1)	0.9 (0.2–5.6)
Treatment	No	39/91	42.9	1.0 (Reference)	
	Yes	15/32	46.9	1.2 (0.5–2-6)	0.4 (0.1–1.2)

^a^Based on 115 patients in the multivariate regression

## Discussion

This study, investigating the association between *COMT* haplotypes and recovery after whiplash injuries, could not detect any differences in the recovery rate between the three *COMT* haplotypes. Nor were any significant differences detected between *COMT* haplotypes regarding the secondary outcome measures, i.e., level of pain and quality of life according to the SF-36 at 12 months. However, the multivariate analysis showed that high levels of pain and anxiety after the whiplash injury were significantly associated with non-recovery.

In a meta-analysis by Tammimäki et al. of pain and psychological symptoms after motor vehicle collisions, an association was found between different *COMT* haplotypes and musculosceletal pain [12]. Furthermore, they noted that several other factors, such as pain stimulus, chronicity of pain, sex, sampling and sample size, were identified as contributors to differences in their results. McLean and co-workers [11] reported on pain and psychological symptoms after motor vehicle collisions and found a connection with the pain sensitive *COMT* genotype. In our study, pain and anxiety affected the outcome, but were not connected to the *COMT* haplotypes. It is possible that the finding that 20% of the respondents reported symptoms of anxiety and 9% had signs of PTSD prior to the accident affected the results. It has previously been shown that anxiety and PTSD symptoms can moderate pain sensations [29, 30].

Another explanation for the findings in this study, as compared to previous research, could be the differences in ethnicity between the studies. Mclean et al. reported differences in pain perception in a European-American population [11], Xiang et al. could not detect any differences in pain perception in a Chinese population [31], while, in the present study, almost 90% of the participants consists of Caucasians. One limitation of this study is the lack of a control group. The distribution of *COMT* haplotypes varies between different settings and geographic locations [8, 32] and may affect the outcome. Furthermore, gender differences are also relevant in this field of research [33]. Indeed, several studies point towards a complex COMT-by-gender interaction effect [34, 35]. However, in our study, no difference could be detected between the sexes, probably due to a small sample size.

Nor could our study demonstrate any differences in pain ratings between patients with the most common *COMT* genotypes. This finding is in contrast to the findings by Bortsov and co-workers, who found differences between genotypes 6 weeks after a motor vehicle collision [36]. One reason might be the different methods of *COMT* haplotyping and subgrouping compared to that cohort. Grouping into three haplotypes (i.e., LPS; APS; HPS) was done as suggested by Diatchenko and colleagues [15]. Another reason for the contradictory findings might be differences in self-reporting methodology (verbal vs. VAS) and the timing for rating pain. In our study, the pain ratings were done within two weeks after the accident, a time point at which the level of pain still might not have been stabilized and the frequency of high pain ratings were high and, consequently, could lead to a poorer outcome [4, 37].

Pain recovery comprises a complex interplay between several systems, from sensory to immune to brain circuits [38]. Research on genetic susceptibility to pain has identified several *COMT* SNPs as possible candidates; however, more studies are needed to define the possible causality of this relationship.

## Conclusion

In summary, no relationship between the outcome regarding reported non-recovery or pain levels and *COMT* haplotypes in patients with acute whiplash injuries could be detected in this material. Nor did we find associations between psychological factors and the *COMT* haplotypes. High levels of initial pain and anxiety were associated with non-recovery, thereby confirming previously published literature.

## Abbreviations

APS: Average Pain Sensitivity
COMT: Catechol-O-Methyl Transferase
HPS: High Pain Sensitivity
LPS: Low Pain Sensitivity
PTSD: Posttraumatic Stress Disorder
SNP: Single Nucleotide Polymorphism
VAS: Visual Analogue Scale
WAD: Whiplash-Associated Disorder
